# Intensity-modulated radiotherapy with integrated-boost in patients with bone metastasis of the spine: study protocol for a randomized controlled trial

**DOI:** 10.1186/s13063-018-2452-7

**Published:** 2018-01-22

**Authors:** Tanja Sprave, Stefan Ezechiel Welte, Thomas Bruckner, Robert Förster, Tilman Bostel, Ingmar Schlampp, Nils Henrik Nicolay, Jürgen Debus, Harald Rief

**Affiliations:** 1 Department of Radiation Oncology, University Hospital Heidelberg, Im Neuenheimer Feld 400, 69120 Heidelberg, Germany; 2 Department of Medical Biometry, University Hospital Heidelberg, Im Neuenheimer Feld 305, 69120 Heidelberg, Germany; 3 National Center for Radiation Oncology (NCRO), Heidelberg Institute for Radiation Oncology (HIRO), Im Neuenheimer Feld 400, 69120 Heidelberg, Germany; 4 Department of Radiation Oncology, Heidelberg German Cancer Research Center (DKFZ), Heidelberg, Germany

**Keywords:** Bone metastases, IMRT, SIB, Palliative radiotherapy

## Abstract

**Background:**

Stereotactic body radiation therapy (SBRT) using intensity-modulated radiotherapy (IMRT) with dose escalation by simultaneous integrated boost (SIB) can be a safe modality for treating spinal bone metastases with enhanced targeting accuracy and improve local tumor control.

**Methods/Design:**

This is a single-center, prospective, randomized, controlled trial. One hundred and twenty patients with spinal bone metastases will receive palliative radiation therapy at the Heidelberg University Hospital. SBRT will be given in five or ten fractions with or without SIB. Four treatment arms are planned: IMRT with 30 Gy in ten fractions, IMRT with 30 Gy in ten fractions and SIB to 40 Gy, IMRT with 20 Gy in five fractions, and IMRT with 20 Gy in five fractions and SIB to 30Gy in five fractions will be compared. The target parameters will be measured at baseline level and at three and six months after radiation.

**Discussion:**

The primary endpoint of this study was to assess and compare the local tumor control (by means of different fractionation schedules and biological doses to the tumor area). Secondary endpoints are acute and chronic adverse events, pain relief, quality of life, and fatigue.

**Trial registration:**

ClinicalTrials.gov, NCT02832765. Registered on 27 July 2016.

**Electronic supplementary material:**

The online version of this article (10.1186/s13063-018-2452-7) contains supplementary material, which is available to authorized users.

## Background

Two-thirds of all cancer patients develop bone metastases during the course of their disease [1]. This form of distant relapse occurs from many types of solid cancers, especially from lung, breast, and prostate [2]. Bone involvement can also be extensive in certain hematologic malignancies [3, 4]. About 30% of all skeletal metastases and 10% of all primary bone tumors are located in the spinal column [5], mostly the lumbar (52%), thoracic (36%), and cervical (12%) spine [6]. The exact mechanism of bone metastasis is not fully understood. It is postulated that bone metastases arise as a detachment of tumor cells from the primary tumor and reach the bone by intravascular penetration, resulting in adhesion in distinct bone areas. The tumor cells cause local changes of the bone structure, caused by an imbalance of bone remodeling. Bone metastases present as osteoplastic, osteolytic, or mixed osteolytic/osteoplastic tumor masses. Many metastatic bone lesions cause few or no symptoms, but for skeletal-related events, pain is the most common symptom; many patients with bone metastasis experience significant pain at some point during their disease course [7]. Other skeletal-related events include reduce activity in daily life (ADL), decreased energy, hypercalcemia, and risk of pathological fractures and neurological deficits. Pathologic bone fractures occur in 5% and spinal cord compression in 10–15% [5]. Restrictions of movement of the spinal column may have a devastating long-term impact on function, mobility, independence, health, and quality of life (QoL). External beam radiotherapy has an established role in the management of patients with bone metastases of the spine. The fractionation schedule applying 30 Gray in ten fractions is a safe and effective treatment modality commonly used to achieve palliation of pain associated with spinal metastases [8]. In about 50–80% of the patients, a decrease of pain is reported and about 30% of the patients showed complete remission of pain [8]. Stereotactic body radiation therapy (SBRT) using intensity-modulated radiotherapy (IMRT) can be a safe treatment option for treating spinal metastasis with enhanced targeting accuracy [9, 10]. Published data report that IMRT to the spine was well tolerated, had no significant late toxicities, and spared other organs at risk [11]. Pre-treatment megavoltage computed tomography (CT) allows for the control of the patient position and improves treatment accuracy [12].

This study aims at evaluating therapeutic outcomes in terms of local control through a higher biological dose in the tumor area by using SIB-IMRT and to test shortened fractionation schedules in palliative patients. Toxicity, fatigue, and QoL improvements will be evaluated. To the best of our knowledge, no comparable randomized study has been described in the literature so far.

## Methods/Design

This is a prospective, randomized, single-center, controlled, exploratory intervention study with four parallel treatment groups aimed at comparing local control rates after RT with and without SIB in patients with bone metastases of the spine. Four different schedules will be evaluated: IMRT with 30 Gy in ten fractions to the whole vertebral body, IMRT with 30 Gy in ten fractions to the whole vertebral body with the application of a SIB to 40 Gy, 20 Gy in five fractions to the whole vertebral body or IMRT with 20 Gy in five fractions to the whole vertebral body with the application of a SIB to 30 Gy. Before enrolment in the study, patients will receive CT and if necessary magnetic resonance imaging (MRI)-based staging examinations to measure the spinal cord dimension. If intraspinal metastases are suspected, an MRI examination is performed beforehand.

RT is performed in systemic/targeted therapy free interval. Radiation therapy will be applied in five fractions per week (Monday to Friday).

**Fig. 1:**
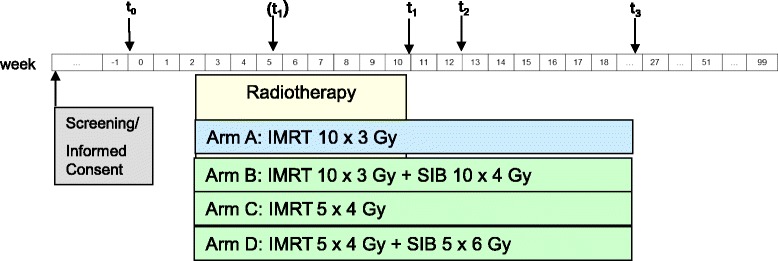
*Flow chart* of IRON-2 trial; t0 = randomization, t1 = end of RT, t2 (3 months after RT) = restaging, t3 (6 months after RT) = restaging

**Fig. 2:**
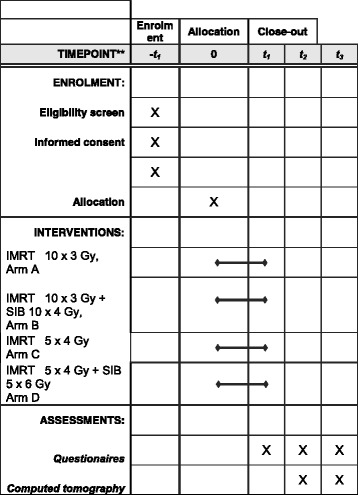
Timeline IRON-2: RT radiotherapy, IMRT intensity-modulated radiotherapy

After recording the baseline measurements, patients will be randomly assigned to one of the four groups. Target parameters will be measured and recorded at baseline (t_1_), at week 12 (t_2_), and again at week 24 (t_3_) (Fig. 1). Follow-up measurements are scheduled to take place at 12, 18, and 24 months after the completion of RT (Fig. 2).

### Recruitment and randomization

The patients will be given all the relevant information about the study by the radiation oncologist at their first presentation to our department (approximately 1–2 weeks before the start of RT). If patients are interested in participating in the study, they will receive a second appointment in order to further inform them and to obtain informed consent. A block randomization procedure will be used to ensure even distribution of patients into the four groups. The randomization procedure will be carried out by a central office. The study personnel responsible for the recruitment and patient care will have no access to the randomization information and the study director will have no influence on the recruitment of patients. The recruitment phase will be concluded with the attainment of the planned number of patients (120 patients in total, 30 patients in each group). Projected recruitment time will be 12 months, and recruitment is scheduled to start in February 2017. Regular study follow-up will end two years after enrolment or, where applicable, with the respective patient’s death.

### Inclusion criteria

- Patients with histologic confirmation of a tumor disease, demonstrating metachronic solitary or multiple metastatic bone lesions of the thoracic or lumbar spine or the sacral bone
- Indication for RT of the bone metastases
- MRI-based staging examinations (if necessary)
- Age 18–85 years
- Karnofsky Index ≥ 50% [13]
- Signed declaration of informed consent

### Exclusion criteria

- Significant neurological or psychiatric disorders, including dementia and epileptic seizures
- Patients with myeloma, lymphoma, or sarcoma
- Previous RT to the treatment area
- MSCC
- Other severe disorders that may prevent the patient’s participation in the study
- Diminished legal capacity
- Any medical of psychological condition that the study director considers a preventive factor for the patient’s ability to complete the study or to adequately understand the scope of the study and to give his/her consent

### Assessment of the primary and secondary endpoints

The aim of the study is to evaluate local control rates in metastatic bone disease. The primary endpoint is defined by bone remodeling according to density in the metastasis. Bone density will be measured at the irradiated region at baseline and at the three-month time-point after the completion of RT (t_2_). Furthermore, the local control is assessed by CT imaging taken before and at three and six months after radiation treatment. Secondary endpoints are vertebral stability, QoL, fatigue, pain, overall survival, bone survival, pathological fracture, and neurological deficits. The baseline examination will be carried out immediately before the start of therapy and is scheduled to comprise the comprehensive recording of the socio-demographic data, the recording of the current pain situation (VAS score), the extent of fractures, the QoL, and the current degree of fatigue. The follow-up examinations will take place after the end of RT, as mentioned above (Fig. 1).

The secondary endpoints such as overall survival, fatigue, QoL, and anxiety will be recorded by using validated questionnaires (EORTC QLQ FA13 [14], EORTC QLQ BM22 [15], and the questionnaire to record stress in patients with cancer [FBK] according to Book et al. [16]). All patients will also be asked to record their pain history by using a pain diary (documentation of medication daily during treatment and once weekly after the completion of treatment, VAS pain scale). The pain response is documented on the VAS (range of 0–10). Complete response is defined as VAS score of 0/10 after three or six months and partial response is defined as a reduced value by at least 2 points after three or six months, according to the international consensus response categories by Chow et al. [17]. Overall survival is defined as time from initial diagnosis until death and bone survival is defined as time from initial diagnosis of irradiated spinal bone metastasis until death (Additional file 1).

### Radiotherapy

Treatment will be planned three-dimensionally using CT scan with a 3-mm slice thickness taken across the involved vertebral region. Immobilization is ensured with an Aquaplast head mask (Aquaplast Corporation, Wyckoff, NJ, USA), vacuum mattress, and Wingstep® (Elekta, Stockholm, Sweden). On the basis of the planning CT, organs at risk will be contoured. The spinal cord is contoured on the basis of visible target on the CT scan with the help of fusion with the MRI data.

The SIB includes the macroscopic tumor. Gross tumor volume (GTV) includes the metastasis. GTV plus 3-mm safety margin gives CTV. CTV corresponds to planning target volume (PTV).

The GTV includes the macroscopic metastatsis. The CTV covers the entire vertebral body including posterior structures (pedicles, processi, and arcus). The CTV with an addition of 3 mm produces PTV. The PTV includes the tumorous vertebra will be covered by the 90% isodose.

Radiation will be applied as IMRT (tomotherapy or step-and-shoot IMRT or volumetric modulated arc therapy [VMAT], energy of 6 MV) using 30 Gy in ten fractions, or 30 Gy in ten fractions and SIB to 40 Gy in ten fractions, or 20 Gy in five fractions, or 20 Gy in five fractions and SIB to 30 Gy. Tolerance doses of the organs at risk are based on the data published for the RTOG 06-31 study [18].

### Therapy drop-out criteria

- Patient’s wish
- Medical condition requiring the discontinuation of therapy in the opinion of the study director or patient
- Insufficient compliance

In case of early drop-out from the study, the planned follow-up scheme will be continued. For non-evaluable patients, the reason will be recorded in the case report forms. Study patients will have the opportunity to refuse individual investigations.

### Statistical analysis

Due to the explorative character of this study, it was not possible to estimate the total number of cases; with a scheduled number of 30 patients per group, it will, however, be possible to detect a standardized mean-value effect (Cohen’s d) of 0.74 with a power of 80% and a significance level of 5%, if a one-factorial variance analysis is calculated. All variables will be analyzed descriptively by tabulation of the measures of the empirical distributions. In case of continuous variables mean values, standard deviations, median, minimum, and maximum values will be documented. Categorical variables will be documented as occurrence and percentage. The primary endpoint will be measured by CT. Results will be presented at three months after completion of RT and differences between groups will be calculated using a one-factorial variance analysis. Differences of categorical variables will be calculated with the Chi-square test. The survival analysis will be performed using the Kaplan–Meier method and log-rank test.

All statistical analyses will be carried out descriptively using SAS software Version 9.4 or higher (SAS Institute, Cary, NC, USA).

### Ethical issues, information, and safety

The study protocol, patient information sheet, and informed consent forms were submitted to the Independent Ethics Committee of Heidelberg University. Approval was given in 2016 (#S-483/2016). Additional recommendations were provided by the committee of experts of the radio-oncology organization in Germany. The study directors will immediately notify the Ethics Committee of all changes made in the study protocol that may have an impact on the safety of the patients. Furthermore, the Ethics Committee will also be notified of all severe adverse events reported to the study directors and of the regular or premature termination of the study. The procedures described in the submitted study protocol regarding the performance, evaluation, and documentation of this study has been selected in such a way that the principles of the Good Clinical Practice guidelines are observed. The regulations regarding medical confidentiality and data protection are fulfilled.

## Discussion

Palliative therapy aims to relieve suffering during all stages of disease and does not have to be limited to end-of-life care. Despite advances in palliative therapies, cancer is still a great burden for patients and painful vertebral bone metastases can strongly reduce the patients’ wellbeing. Limitations of QoL, such as posture changes, reduced movement and pain, fractures, and immobility, can be consequences of vertebral metastases. Therefore, improved QoL in this palliative situation is of pivotal importance. RT has been established as an effective local treatment for vertebral metastases. Therapy goals are a reduction in pain and fatigue, improvement of QoL, prevention of pathological fractures, or neurological deficits.

For patients with vertebral metastases, dose escalations may result in improved tumor control. Since the standardized irradiation volume comprises the spinal cord, it is not possible to reduce radiation doses to the spinal cord even with modern technologies such as IMRT. Adding a SIB to the treatment may result in significant dose reductions in the spinal cord compared to conventional IMRT. Recent trials tested 30 Gy in ten fractions and 40 Gy in five fractions to bone metastases respectively, but these doses are most likely insufficient regarding local tumor control. Therefore, our hypothesis holds that a SIB to the bone metastasis might overcome this problem. A previously published planning study by Lee et al. has shown that IMRT can deliver the concomitant hypofractionated regime proposed and offer benefits in dose delivery. IMRT and VMAT are currently preferred for their superior pre-treatment verification results and shorter planning times [19]. Potential influence on the local control rate of the different IMRT techniques will be investigated in this study. Specifically, the differences in conformity and homogeneity of helical IMRT vs step-and- shoot or VMAT-IMRT techniques are examined in a subgroup analysis.

SBRT has been shown in prospective trials and numerous case series to be safe and effective for the treatment of spinal bone lesions as well as primary bone tumors. One comparison of the palliative efficacy and cost-effectiveness of external beam RT compared with SBRT as a primary treatment for spinal metastases found that patients treated with external beam RT had increased acute toxicities and there was a higher likelihood of them requiring further intervention at the treated sites. A recent trial showed that SIB-IMRT could be successfully applied to vertebral metastases with spinal cord compression in up to four consecutive vertebrae. Good ADL preservation and pain control were achieved with acceptable toxicity [20]. Lubgan et al. demonstrate excellent local control rate of 93% after 24 months in 33 patients who underwent SBRT with SIB. No radiation myelopathy occurred [21]. Nevertheless, the optimal dose schedule could not be found and no prospective trial has been investigated in this setting so far.

### Trial status

Patient recruitment not completed.

## Additional file

Additional file 1: SPIRIT 2013 Checklist: Recommended items to address in a clinical trial protocol and related documents. The additional SPIRIT 2013 Checklist file shows this in more detail. (DOC 121 kb)

## Abbreviations

CT: Computer tomography
CTV: Clinical target volume
GTV: Gross tumor volume
Gy: Gray
IMRT: Intensity-modulated radiotherapy
MSCC: Metastatic spinal cord compression
PTV: Planning target volume
SIB: Simultaneous integrated boost
VMAT: Volumetric modulated arc therapy
